# Supplementary material for: Determinants of adolescents’ Health-Related Quality of Life and psychological distress during the COVID-19 pandemic
Source: PLoS One. 2022 Aug 11;17(8):e0272925. doi: 10.1371/journal.pone.0272925 (PMC9371327; doi:10.1371/journal.pone.0272925)
Supplement: S1 Table — N = 225. (PDF) [file pone.0272925.s001.pdf]

Table S1. Risk factors for adolescents' self- and parent-reported low Health-Related Quality of Life (HRQoL), minimally and fully adjusted models. N=225

	**Adolescent-reported HRQoL (KIDSCREEN-10)**		**Parent-reported HRQoL (KINDL®)**	
	**Low HRQoL (aOR [95%CI])**		**Low HRQoL (aOR [95%CI])**	
	**Minimal model**[1,2]	**Full model** [3]	**Minimal model**[1,2]	**Full model** [3]
**Age of the adolescent (years)**[1]	0.75 (0.54 -1.01)*	0.83 (0.54-0.97)*	0.94 (0.67 - 1.34)	0.95 (0.63-1.41)
**Age of the parent (years)**[1]	1.02 (0.93-1.10)	1.02 (0.89-1.12)	1.01 (0.93 - 1.1)	1.02 (0.94-1.10)
**Sex of the adolescent**[1]				
Boy	1	1		1
Girl	1.45 (0.76-2.76)	1.10 (0.59-2.22)	3.92 (1.59 - 9.69)**	4.31 (1.63-11.10)**
**Self-perceived mood of the parent**[2]				
Good	1	1		1
Average to poor	1.73 (0.71- 4.21)	1.30 (0.46-5.56)	1.59 (0.55 - 4.66)	1.63 (0.51-5.26)
**Financial situation of the household**[1]				
Good	1	1		1
Average to poor	1.75 (0.72-4.07)	2.30 (0.85-6.23)	1.56 (0.59 - 4.13)	1.36 (0.56-5.65)
No answer	0.64 (0.04- 7.04)	0.53 (0.07-5.27)	0.49 (0.06 - 3.96)	0.67 (0.07-6.67)
**Household size (individuals)**[1]	1.10 (0.72-1.67)	1.52 (0.79-2.66)	1.12 (0.79 - 1.59)	1.20 (0.79-1.78)
**Household density**[1]				
Non-crowded	1	1		1
Crowded	2.39 (0.75-7.59)	2.08 (0.48-6.70)	2.68 (0.81 - 8.85)	2.05 (0.64-6.61)
**Change in social media habits**[1]				
Same or less	1	1	1	1
More	1.53 (0.82-2.88)	1.60 (0.92-3.70)	0.81 (0.35 - 1.87)	0.83 (0.62-1.45)
**Screen time (hours)**[1]	0.97 (0.83-1.14)*	0.89 (0.79-0.98)*	0.94 (0.75 - 1.19)	0.95 (0.82-1.06)
**Parent anti-SARS-CoV-2 serology**[2]				
Negative	1	1	1	1
Positive	2.60 (1.05-9.30)*	3.33 (1.20-9.12)*	2.29 (0.91 - 5.73)	2.01 (0.69-6.34)
**Adolescent anti-SARS-CoV-2 serology**[2]				
Negative	1	1	1	1
Positive	1.22 (0.42 -3.57)	1.08 (0.57-2.27)	1.84 (0.72 - 4.74)	1.38 (0.44-4.03)
**Parents' marital status**[1]				
Married or in couple	1	1	1	1
Divorced, separated, single or widowed	0.97 (0.34-3.01)	0.84 (0.27-2.20)	0.77 (0.26- 2.78)	0.57 (0.16- 2.13)

Results are adjusted odds ratios (aOR) and 95% confidence intervals (CI) from multivariable generalized estimating equations. aOR of continuous variables applicable for each additional unit.

* indicates P < 0.05; ** indicates P < 0.01

[1] Minimal model adjusted for age and sex; [2] Minimal model adjusted for age, sex, financial situation and household density;

[3] Full model adjusted for all covariates in the first column
