# Supplementary material for: Determinants of adolescents’ Health-Related Quality of Life and psychological distress during the COVID-19 pandemic
Source: PLoS One. 2022 Aug 11;17(8):e0272925. doi: 10.1371/journal.pone.0272925 (PMC9371327; doi:10.1371/journal.pone.0272925)
Supplement: S2 Table — (N = 225). (PDF) [file pone.0272925.s002.pdf]

Table S2. Risk factors for adolescents' self-reported loneliness and sadness, and parent-reported low emotional well-being, minimally and fully adjusted models. (N=225)

	**Self-reported sadness**		**Self-reported loneliness**		**Parent-reported low emotional well-being (KINDL®)**	
	**Sadness (aOR [95%CI])**		**Loneliness (aOR [95%CI])**		**Low emotional well-being (aOR [95%CI])**	
	**Minimal model[1,2]**	**Full model [3]**	**Minimal model[1,2]**	**Full model [3]**	**Minimal model[1,2]**	**Full model [3]**
**Age of the adolescent (years)[1]**	1.02 (0.79 - 1.31)	0.96 (0.72-1.13)	1.11 (0.84 - 1.46)	1.01 (0.80-1.23)	0.87 (0.66-1.16)	0.84 (0.62-1.17)
**Age of the parent (years)[1]**	1.06 (1.01 - 1.13) *	1.06 (1.00-1.13) *	1.04 (0.98 - 1.11)	1.04 (0.97-1.12)	1.08 (1.01-1.17)	1.10 (0.97-1.22)
**Sex of the adolescent[1]**						
Boy		1		1		1
Girl	2.75 (1.61 - 4.83) **	3.20 (1.67-6.16) **	3.51 (1.79 - 6.91) **	4.10 (1.92-8.78) **	2.49 (1.23 -5.04) *	2.73 (1.22-6.06) *
**Self-perceived mood of the parent[2]**						
Good		1		1		1
Average to poor	2.99 (1.31 - 6.82) *	2.62 (1.10-6.23) *	1.77 (0.71 - 4.44)	1.68 (0.71-3.98)	0.91 (0.33-2.51)	0.94 (0.39-2.30)
**Financial situation of the household[1]**						
Good		1		1		1
Average to poor	2.31 (1.11 - 4.81) *	2.31 (1.01 - 6.10) *	1.98 (0.84 - 4.46)	1.98 (0.68-4.63)	2.16 (0.98-6.20)	2.37 (0.98-7.38) *
No answer	0.67 (0.2 - 2.29)	0.85 (0.30-2.91)	1.01 (0.21 - 3.8)	1.41 (0.36-5.50)	0.99 (0.20-3.21)	0.98 (0.29-4.76)
**Household size (individuals)[1]**	1.01 (0.79 - 1.29)	1.15 (0.97-1.48)	1.03 (0.77 - 1.38)	1.17 (0.67-1.59)	0.79 (0.53-1.17)	0.73 (0.39-1.21)
**Household density[1]**						
Non-crowded		1		1		1
Crowded	1.97 (0.74 - 5.25)	1.60 (0.58-4.77)	1.03 (0.32 - 3.32)	0.99 (0.74-1.65)	3.03 (1.20-7.65) *	3.12 (1.10-9.37) *
**Change in social media habits[1]**						
Same or less		1		1		1
More	2.26 (1.26 - 4.04) *	2.07 (1.08-3.97) *	1.92 (0.99 - 3.7)	1.88 (0.96-3.90)	1.41 (0.57-3.48)	1.57 (0.67-3.40)
**Screen time (hours)[1]**	1.09 (0.94 - 1.27)	1.04 (0.91-1.27)	1.08 (0.91 - 1.28)	1.06 (0.87-1.27)	0.95 (0.72-1.08)	0.98 (0.71-1.15)
**Parent anti-SARS-CoV-2 serology[2]**						
Negative		1		1		1
Positive	2.19 (1.10 - 4.35) *	2.55 (1.14-5.67) *	1.52 (0.60 - 2.92)	2.05 (0.84-5.01)	0.94 (0.38-2.46)	0.67 (0.23-1.98)
**Adolescent anti-SARS-CoV-2 serology[2]**						
Negative		1		1		1
Positive	0.87 (0.43 - 1.79)	0.54 (0.22-1.32)	0.30 (0.11 - 0.85) **	0.19 (0.05-0.70) **	1.20 (0.50-2.86)	1.59 (0.57-4.38)
**Parents' marital status[1]**						
Married or in couple	1	1	1	1	1	1
Divorced, separated, single or widowed	1.20 (0.55-2.01)	1.17 (0.61-2.20)	1.86 (0.86-4.03)	1.36 (0.58-3.21)	0.95 (0.53-2.93)	0.84 (0.27-2.20)

Results are adjusted odds ratios (aOR) and 95% confidence intervals (CI) from multivariable generalized estimating equations. aOR of continuous variables applicable for each additional unit.
* indicates P < 0.05; ** indicates P < 0.01
[1] Minimal model adjusted for age and sex; [2] Minimal model adjusted for age, sex, financial situation and household density;
[3] Full model adjusted for all covariates in the first column
